# An Integrated Care Model With Implementation Roadmap to Improve *Chlamydia trachomatis* Management and Control in India

**DOI:** 10.3389/fpubh.2018.00321

**Published:** 2018-11-09

**Authors:** Pierre P. M. Thomas, Ramesh R. Allam, Elena Ambrosino, Jelena Malogajski, Jonathan A. Lal, Servaas A. Morré, Remco P. H. Peters

**Affiliations:** ^1^Department of Genetics and Cell Biology, Faculty of Health, Medicine and Life Sciences, Institute for Public Health Genomics (IPHG), GROW School for Oncology and Developmental Biology, Maastricht University, Maastricht, Netherlands; ^2^SHARE India, Hyderabad, India; ^3^Department of Public Health, School of Health Professions, Long Island University, Brooklyn, NY, United States; ^4^Department of Molecular and Cellular Engineering, Jacob Institute of Biotechnology and Bioengineering, Sam Higginbottom University of Agriculture, Technology and Sciences, Allahabad, India; ^5^Laboratory of Immunogenetics, Department of Medical Microbiology and Infection Control, VU University Medical Center, Amsterdam, Netherlands; ^6^Clinical Care and Research, Anova Health Institute, Johannesburg, South Africa; ^7^Department of Medical Microbiology, CAPHRI School for Public Health and Primary Care, Maastricht University, Maastricht, Netherlands

**Keywords:** *Chlamydia trachomatis*, sexually transmitted diseases, integrated care, sexual and reproductive health, India

## Abstract

*Chlamydia trachomatis* is the world's most prevalent bacterial Sexually Transmitted infection (STI). It is associated with a wide range of health consequences and sequelae in both the short and long term. Enhanced control of urogenital *C. trachomatis* infection is particularly important in low- and middle-income countries such as India, where most of the burden goes unnoticed and where limited systematic data is available to gauge the current situation. The World Health Organization (WHO) recently issued its latest strategy on STIs, which is aligned with the achievement of the Sustainable Development Goals (SDGs). Taking the WHO framework into account; this paper puts forward an integrated care model to strengthen the management and control of *C. trachomatis* in India. The model is compiled of five key components of STI management (awareness, prevention diagnosis, treatment and follow-up). The model considers barriers to effective C. trachomatis control into account. The barriers are discussed and compiled into different categories. A roadmap for the implementation of other similar models to enhance *C. trachomatis* control in the future is provided.

## Introduction

The World Health Organization's (WHO) strategy for sexually transmitted infection (STI) control is summarized in the “Global health sector strategy on Sexually Transmitted Infections (STIs) 2016-2021, toward ending STIs” (1). The strategy is aligned with the Sustainable Development Goals (SDGs) which builds upon the Millennium development Goals (MDGs) and offer the layout for better and more sustainable future for all the countries in the world (2). The focus on sexual and reproductive health has become stronger in the SDGs compared with the MDGs (3). The WHO STI strategy addresses several SDGs, including the 3rd goal of good health and wellbeing, the 5th goal of gender equality and the 10th aiming at reducing inequalities (4). The WHO STI strategy is structured around five strategic directions aimed at ending STI epidemics as major public health concerns (Table 1).

**Table 1:** Strategic directions of WHO strategy on STIs.

**Strategic action**	**Basic components**
Information for focused action	Better understand the STI epidemic Need for systematic data and surveillance
Interventions for impact	First direction of universal health coverage Describe the basic package of high impact interventions
Delivering for equity	Second dimension of universal health coverage Identify the best approaches for delivering the continuum of care to different populations and address the barriers
Financing for sustainability	Third dimension of universal healthcare coverage Identify sustainable and innovative models for financing of STI control response Approaches for reducing costs
Innovation for acceleration	Identify major gaps in knowledge and technologies where innovation is required

The main focus of the strategy is on *Neisseria gonorrhoeae* to prevent and stop spread of drug-resistance, *Treponema pallidum* (prevention of congenital syphilis) and on HPV (reducing cancer-associated mortality). *Chlamydia trachomatis* infection, with an estimated >130 million incident cases worldwide, is recognized as important, but the WHO strategy suggests that there is a lack of efficient interventions to successfully address the global burden of *C. trachomatis* (1, 5, 6).

*Chlamydia trachomatis* infection has a significant impact on sexual, reproductive, maternal and child health. The infection is relatively easily treated with antibiotics (azithromycin and/or doxycycline) that are widely available, even in resource-constrained LMICs (7). Despite the availability of antibiotics many healthcare barriers exist to successful management and control of genital *C. trachomatis* infections in LMICs such as India. Additionally, the implementation of routine diagnosis and syndromic management has not succeeded in effectively addressing the burden. In this paper we discuss current *C. trachomatis* control efforts in India and propose an integrated approach aimed at strengthening health service provision to be inclusive of *C. trachomatis* infections in the Indian setting to include comprehensive care for *C. trachomatis*.

## The burden of *Chlamydia trachomatis* infection in india

India is home to an ethnically diverse population of over one billion inhabitants and to one of the most significant burdens of infectious diseases worldwide. The healthcare system is split between the public healthcare sector, catering to lower-income populations and the private sector mostly utilized by high-income populations (8, 9). It was estimated in 2005–2006 that the private healthcare sector ranks as the first provider of care for 70% of urban and 63% of rural homes respectively (10). There is a large difference in quality of healthcare with the lowest quality of care generally provided in rural public healthcare settings (11). High maternal and child mortality rates emphasizing this large divide (12).

India does not have a surveillance system for STIs other than HIV and there are limited data available with regards to the burden of *C. trachomatis* infections and its impact on health. A recently published (2017) systematic literature identified a limited number (*n* = 27) of studies that assessed the prevalence of *C. trachomatis* in India, with large heterogeneity in terms of prevalence reported and population groups included (13). It should be furthermore stressed that major differences exist within India regarding the genetic makeup of the populations, as well as the functioning of the healthcare system at the state level. All these factors can have in impact of the STI situation. There is no clear overall estimate of *C. trachomatis* prevalence in the Indian population, but studies did show a high prevalence of *C. trachomatis* infection among symptomatic women presenting with vaginal discharge at the outpatient department. Much lower prevalence rates were observed in a community-based sample of Indian women (14, 15). High prevalence of infection (up to 15% based on PCR testing) as well as seroprevalence (up to over 60% using ELISA based serology) is reported for women consulting for sub-fertility, confirming the established link between *C. trachomatis* infection and sub-fertility in the Indian setting (16–21).

India has implemented syndromic case management of STI in the public health sector. This means that individuals with specific symptoms are treated empirically with a combination of antibiotics (22). Currently, vaginal and urethral discharge, the main clinical presentations of *C. trachomatis* infection, is treated with a combination of azithromycin and cefixime. An important limitation of the syndromic approach, in particular in case of *C. trachomatis*, is that asymptomatic infections, so over 80% of all infections, remain untreated. This puts a large number of individuals at risk for long-term sequelae associated with this infection. A recent study from India highlights the poor results of syndromic management as well as its link to over-prescription of antimicrobials and to antimicrobial resistance (23). These findings are corroborated by studies from other developing countries, such as South Africa (24).

**Figure 1:**
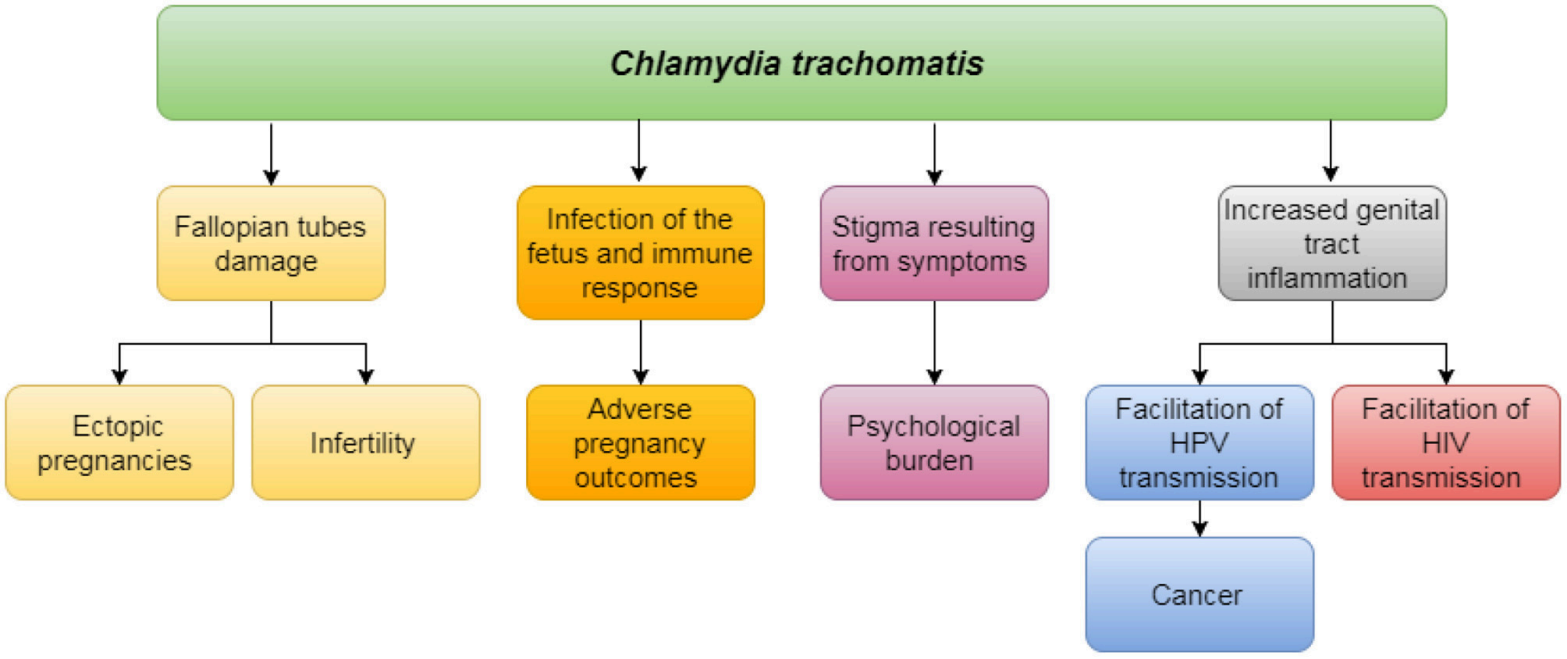
Impact of untreated *Chlamydia trachomatis* on individual's health.

Untreated *C. trachomatis infections*, presenting with or without symptoms, may impact on individual's health through in various ways (Figure 1).

Untreated chlamydial infections may evolve into Reproductive Tract Infections (RTIs), which may lead to tubal factor infertility and pelvic inflammatory disease and increased risk of ectopic pregnancy in women (and subsequent morbidity and mortality) (25–28). *Chlamydia trachomatis* may directly impact pregnancy outcomes, through causing spontaneous abortion and stillbirth, may also result in pre-term labor associated with poor outcomes of neonates (29, 30). Genital tract inflammation facilitates transmission and acquisition of HIV and possibly human papilloma virus (HPV) infection. Finally, there is a significant psychological burden associated with STIs in general, including *C. trachomatis*. Impact on sexual pleasure may negatively affect the individual's sexual health, as well as, relationships. Perceived internal and external stigma and discrimination impact general well-being, especially in conservative societies such as in India (31, 32). The challenge here is that, in the absence of diagnostic texting, these symptoms associated with STI may just as well be caused by any other, not sexually transmitted, infection (33).

## Current initiatives and barriers to *Chlamydia trachomatis* control in india

**Figure 2:**
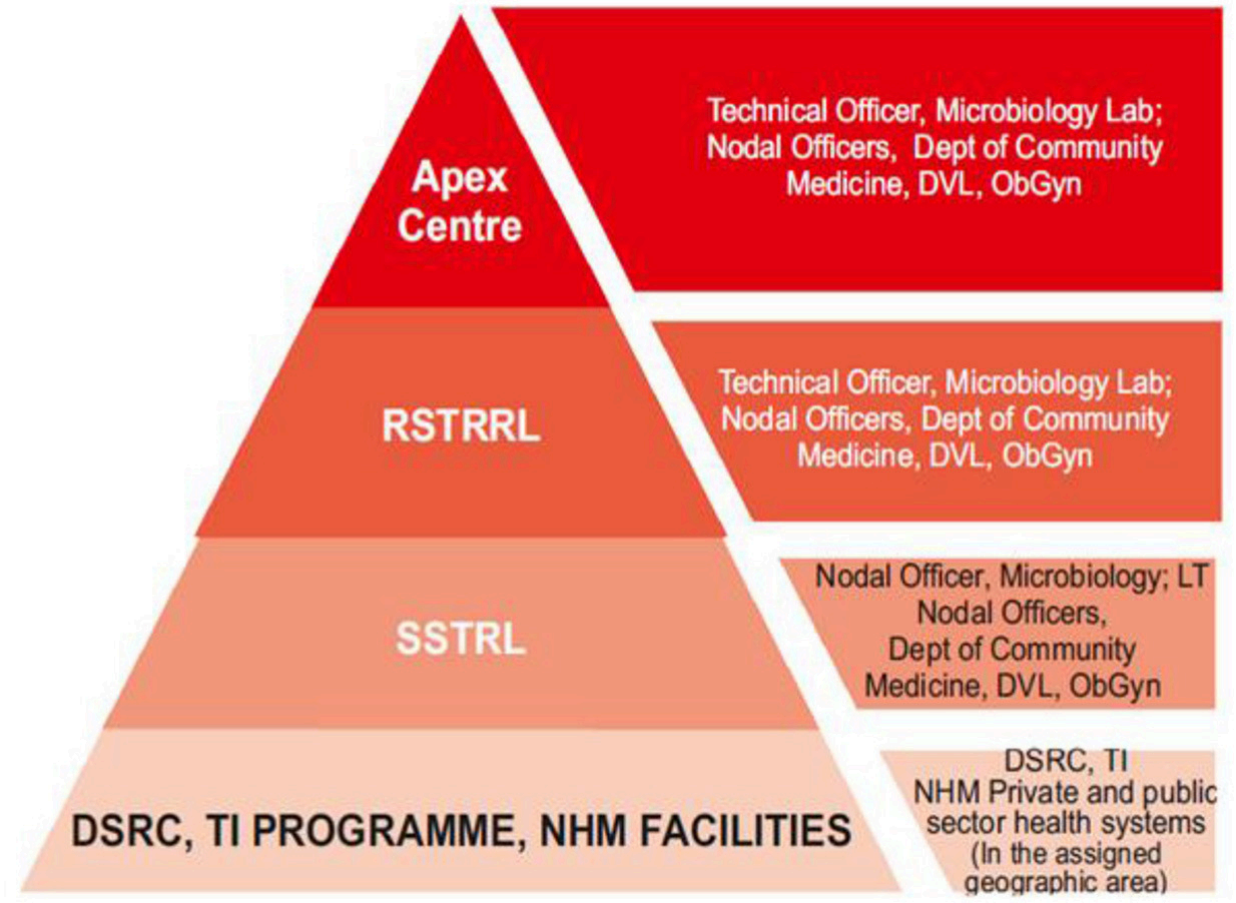
Levels of STI care in India including key actors, adapted from the operational guidelines of the Government of India.

The current situation of STI care in India is challenging: Patients have to circumnavigate across a wide range of providers, both public and private due to the fragmentation of the healthcare delivery system (34). Out-of-pocket expenditures and informal payments are common and constitute a further barrier to patients accessing care (35, 36). The current system put forward by the Government of India is centered around a referral chain where several actors with different roles are active. The referral chain is featured in the diagram below, along with the key actors at each step (Figure 2).

STI clinics and healthcare settings, such as Gynecology and Obstetrics Outpatient departments (OPD) that see patients for these conditions constitute the lowest echelon of the referral chain. The current policy stipulates that syndromic management should be applied to patients presenting with symptoms indicative of STIs, based on pre-defined algorithms. The strategy however encourages laboratory testing whenever it is available. The task of identification and testing of samples gathered at the previously outlined level is the role of the regional and state level laboratories (RSRTL and SSRTl, respectively). These entities should avail the necessary equipment to identify different STIs, including chlamydia and gonorrhea, using PCR or other tests. The regional and state laboratories are also conjointly in charge of monitoring etiological trends and to test for sensitivity to drugs on the samples obtained from public and private STI testing sites. They then ought to report their findings, in order to update syndromic management algorithms when necessary and inform patient treatment (37, 38). The current STI control policy warrants special attentions for core and high-risk groups, as well as, for cases of re-infection and/or treatment failure (22). These activities are overseen at the country level by the national Apex laboratory, located at Safdarjang Hospital in New Delhi which is in charge of overseeing all of the activities related to STI testing and control. There are additional activities undertaken by the government of India aimed at addressing the burden of STIs. A recent project aimed at the eradication of congenital syphilis is also currently in progress and mainly targets pregnant women. In spite of these efforts and endeavors, challenges toward sound management of *C. trachomatis* and other STIs remain. One of the main hurdles is the lack of awareness on SRH-related topic within Indian communities, particularly among youths (39). Cultural and traditional factors also impact negatively on STI control. The conservative cultural beliefs of Indian communities are not benevolent toward the use of STI services particularly among young women (40). Also, there is limited awareness surrounding issues of STIs, and knowledge about STIs other than HIV is low (41, 42). Gender inequality is similarly a compounding factor for both risk and perceived risk of contracting an STI among married women (43, 44). Lack of knowledge of sexual and reproductive health issues among men has also been put forward (45).

The various barriers to accessing services to address *C. trachomatis* infection in India are summarized below (Table 2). These are divided in barriers linked to culture and education and barriers inherent to logistics and resources.

**Table 2:** Barriers to *Chlamydia tracho*matis control.

**Logistics and resources**	**Culture and education**
Availability of diagnostic tests and drugs: Patients should be able to avail reliable tests, on which a diagnostic can be formulated.	Health Seeking behavior: Patients might try to self-medicate or rely upon traditional healers before seeking allopathic care, delaying the start of treatment
Cost of diagnostic tests and drugs: Elevated costs of tests and drugs can severely deter people from seeking treatment	Education and attitude of healthcare professionals: nurses, physicians and other healthcare professionals should possess sufficient knowledge and training to deal with STI-related symptoms in a non-discriminatory fashion. Harsh, judgmental and disrespectful ways have the potential to deter patients from seeking care
Access to Healthcare Infrastructure: Opening hours, waiting lines and informal payments may hinder access to care, especially in settings where healthcare delivery is fragmented	Cultural and educational climate: Patients that are aware of the relevance of their sexual and reproductive health may be more likely to seek treatment. Conversely, Adverse community perceptions of STIs and their association with unethical behavior are restrictions to sustainable management of STIs

## An integrated model to improve *Chlamydia trachomatis* care

One of the important aspects highlighted in the WHO strategy is the implementation of a continuum of care, while adopting a holistic approach, aimed at tackling the important driving forces behind the spread of STIs including *C. trachomatis*. Integrated care is a conceptual framework that conceptualizes the idea of continuum of care theorized in the WHO strategy. It is defined as “*The management of health services so that people get the care they need, when they need it in ways that are user-friendly, achieved desired results and provide value for money*” (46). Important objectives of integrated care are streamlining the provision of services and to help the patients navigate the delivery of care with simplicity. Such an approach would be appropriate to the Indian setting, where healthcare provision is scattered and informal payments are common (34–36). An integrated approach would facilitate access to care, especially for women and in a context of stigma and discrimination (47). Finally, the integrated approach would promote improvement of clinical practices and prudent use of antibiotics, even in settings where STIs are treated syndromically (48, 49). Furthermore, the previously outlined levels of STI care in India constitutes a valid point of entry for the implementation of an integrated care model aimed at *C. trachomatis* control and management. This is further supported by the fact that the current strategy aims at bridging the gap between public and private healthcare delivery and management of *C. trachomatis* treatment. Disease control can in fact be integrated at several levels of the healthcare delivery system. Although there are no accounts of integrated care specifically directed at *C. trachomatis*, there are many accounts of integration of STI services in primary care (50). In such case, healthcare professionals may raise awareness and provide diagnostic testing for STIs, including *C. trachomatis*, as part of their routine package of care. The screening program for HIV and syphilis among pregnant women also constitutes a valid opportunity for integration of *C. trachomatis* diagnosis and management during the same clinic visit, as the chlamydial infections may negatively impact on pregnancy outcomes and neonatal health (51).

**Figure 3:**
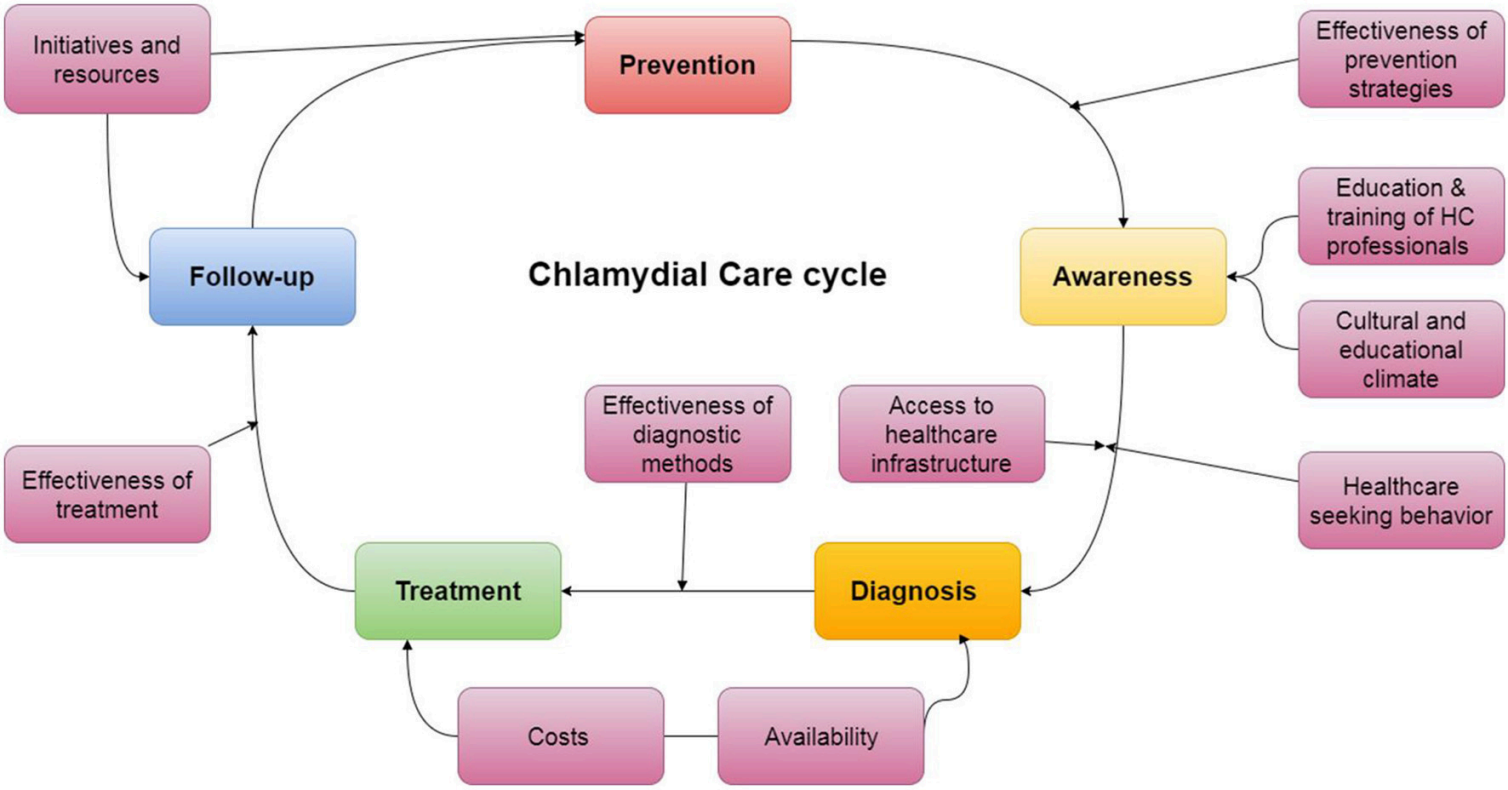
Proposed integrated model to improve *Chlamydia trachomatis* management and control in India.

Based on the theoretical strengths of an integrated model, in line with the WHO strategy, and the barriers and limitations to *C. trachomatis* discussed in the previous paragraph, we propose such an integrated model for continuum of care to improve *C. trachomatis* control in India (Figure 3) (52, 53). The five key components in this model are awareness, prevention, diagnosis, treatment and follow-up. These are linked to factors that influence their delivery and implementation. These components constitute the strategic step in sound *C. trachomatis* management, as all of these steps allow for each infection to be detected, treated and further spread of the infection. They allow patients to benefit from an evidence-based approach and to be empowered in the face of a set of symptoms which may otherwise lead to discrimination. Additionally, this framework emphasizes the links between the different actors, and well as the factors that contribute to the success of such a model and presents an opportunity for an ongoing feedback loop for quality improvement. The combination of these tasks may however result in additional workload for the care personnel. Performing each of these actions moreover necessitates logistic efforts in order to ensure the functioning of the model. This requires political commitment to the issue of *C. trachomatis, and* overall support from the community. The implementation process and delivery of integrated care need to be close monitored as specific interventions and contextual adaptation may be required during the process to achieve success. Also, full commitment of relevant stakeholders is imperative and needs to be brought in from the earliest possible stage of implementation. Many lessons can be drawn from the implementation of integrated care frameworks in other LMICs, such as South Africa. This country has seen integration of STI care at different levels of healthcare delivery, and the results stress the importance of quality monitoring and, evaluation of these projects (54, 55).

## Steps for the future

**Figure 4:**
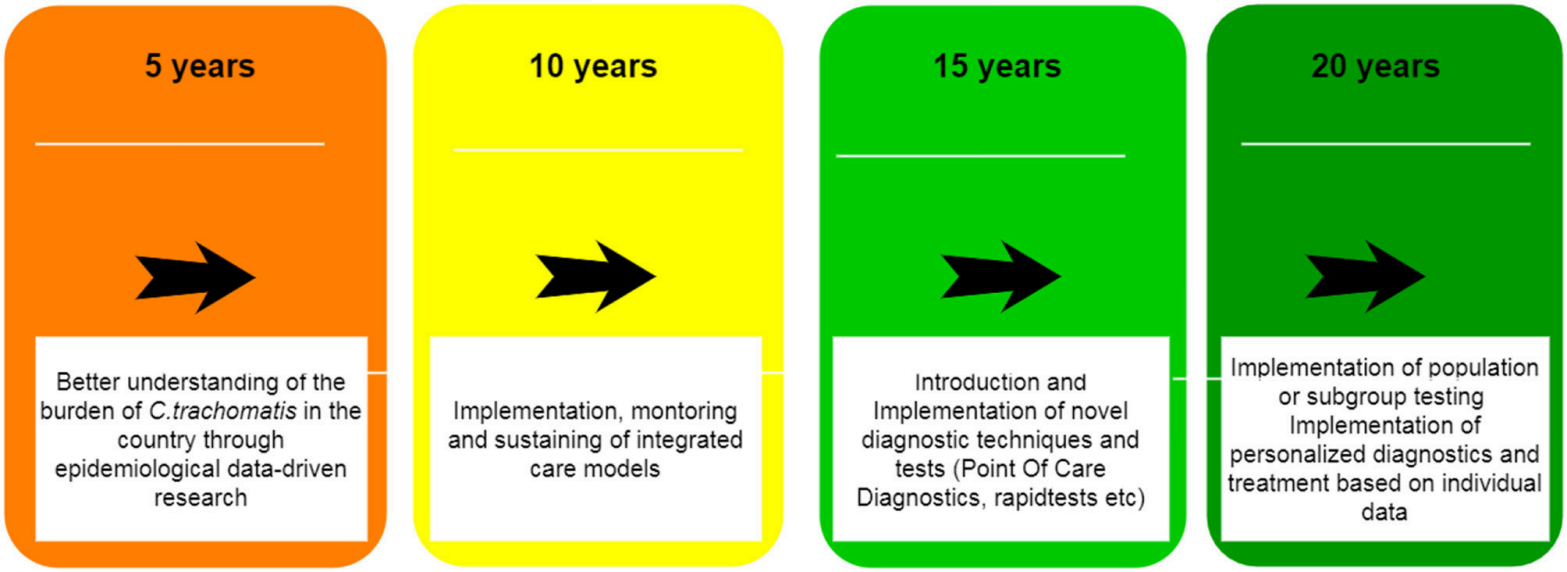
A roadmap to improve *Chlamydia trachomatis* management and control in India over the next 20 years.

STIs including *C. trachomatis* often fail to get much attention and resources from health policy makers to effectively deal with the burdens of infection in developing countries, such as India. To allow for more specific discussions to improve *C. trachomatis* control, a clear implementation plan with timeframe is required. We propose a tentative set of goals based on current state of research, translation and implementation to improve *C. trachomatis* control in Indian in the next 20 years (Figure 4).

The first important step is to better understand the burden of *C. trachomatis* infections in India, including specific risk groups in the populations and attributed risk factors. This translates into a need for data at different levels. First a structure for systematic and routine gathering of clinical data should be articulated. This could take the shape of sentinel surveillance of symptoms or clinical cases confirmed in the lab a selection of (a)symptomatic populations. Hospitals and healthcare institutions that are in the possession of databases and biobanks also ought to be encouraged to share their information and potentially open it for research purposes (56). Studies aiming at evaluation incidence and prevalence of the disease in communities and specific population groups (Commercial sex workers, Men who Have sex with Men) should be initiated and supported. The authors have obtained a Dutch grant; acronym “*ChlamIndia*,” to investigate this and first results will be obtained in the beginning of 2018. Data on the utilization of specialized sexual and reproductive health care also would need to be gathered. In addition, calls for systematic reviews of the literature and meta-analyses of the existing data ought to be performed. These data are imperative to successful design and implementation of control strategies.

The second step would translate into the implementation of an integrated care model, such as proposed in the previous paragraph. This would have to be accompanied by constant monitoring of performance and quality of implementation of these models and a supported managerial and political climate.

The third step caters for the widespread introduction of diagnostic tests to improve clinical care and to start detecting and addressing the burden of asymptomatic *C. trachomatis* infections in both public and private sector. Availability of a point-of-care (POC) tests that provide quick result and allow for same-day treatment, instead of shipment of specimens to the laboratory and the need for a return visit to discuss the results, would be highly supportive of this process (57, 58). Such tests are already available for some STIs, yet they presently are still underperforming when compared to international NAAT standards as performed in the laboratory especially for *C. trachomatis* (59). Self-sampling procedures have also been explored in other LMICs and have helped to reduce the barriers toward testing (60). This technique could also help address issues in the Indian setting.

The final step evolves around the principle of precision medicine and constitutes implementation of individually tailored diagnostic and treatment procedures into routine practice such as Point of Care diagnostics and mobile tools. For example, some molecular variations and single nucleotide proteins have been linked to higher likelihood of infection with *C. trachomatis* or with the development of its long-term sequelae, such as infertility (61). The implementation of genetic and genomic information into routine clinical practice would enable a precision approach to each single case and every single patient, allowing for the full spectrum of information to be taken into account (62). This would contribute to effectiveness and sustainability of services, while providing an empowering environment for the patients to seek care. Although these principles have great promise, a large part of technical development and improved understanding of microbiological, immunological, and host-genetic factors of *C. trachomatis* infection is required before this will be the new “standard practice.”

## Conclusion

*Chlamydia trachomatis* is the most prevalent bacterial STIs in the world and it is associated with a wide-range of sequelae. Efforts that support management and control of *C. trachomatis* infection are imperative to improve health outcomes in Low and Middle Income Countries in line with the Sustainable development goals. The challenges surrounding *C. trachomatis* control in India that we presented are characteristic for many LMICs. Bold efforts are warranted to address the burden of disease and underlying healthcare, cultural and traditional barriers. We have proposed an innovative integrated model alongside an implementation roadmap that would support enhance *C. trachomatis* management and control in India. This provides a blueprint for debate that ultimately aims to improve health outcomes in resource limited settings across the globe.

## Author contributions

Primary research and models were developed by PT, under close supervision of RP and SM. The manuscript was compiled by PT and steadily improved based on comments from RP and SM. Further comments and advice on the future steps and precision medicine approach were provided by EA and JM. Advice, and insights from the Indian perspective were provided by JL. Information on the current situation and strategies in India, as well an overview of the current policies was provided by RA.

### Conflict of interest statement

The authors declare that the research was conducted in the absence of any commercial or financial relationships that could be construed as a potential conflict of interest. The reviewer GS and handling editor declared their shared affiliation at time of review.
